# Supplementary material for: Ethnomedicinal appraisal of plants used for the treatment of gastrointestinal complaints by tribal communities living in Diamir district, Western Himalayas, Pakistan
Source: PLoS One. 2022 Jun 8;17(6):e0269445. doi: 10.1371/journal.pone.0269445 (PMC9176800; doi:10.1371/journal.pone.0269445)
Supplement: S1 Table — (PDF) [file pone.0269445.s001.pdf]

Local Name	Taxonomic Name	Family
Zoon	*Artemisia maritima* L./RW735	Compositae
Philil	*Mentha longifolia* (L.) L./RW745	Lamiaceae
Nerlay Zoon	*Tanacetum faconeri* Hook.f./RW739	Compositae
Philil	*Mentha piperita* L./RW736	Lamiaceae
Kasho/Paloan	*Allium cepa* L./RW744	Amaryllidaceae
Choro	*Pimpinella diversifolia* DC. /RW746	Apiaceae
Teetar	*Hylotelephium telephioides* (Ledeb.) H. Ohba/RW741	Crassulaceae
Chontal	*Rheum webbianum* Wall./RW747	Polygonaceae
Nerlay Churki	*Oxyria digyna* (L.) Hill/RW740	Polygonaceae
Lilio	*Viola serpens WalL.*/RW748	Violaceae
Shey Lamay	Persicaria amplexicaulis (D.Don) Ronse Decr./RW749	Polygonaceae
Cheti Char	*Cichorium intybus* L./RW750	Compositae
Aaro	*Prunus persica* L./RW751	Rosaceae
Angrezi phang	*Ficus carica* L./RW752	Moraceae
Joi	*Prunus armeniaca* L./RW753	Rosaceae
Love	*Cucumis sativus* L./RW754	Cucurbitaceae
Tumurum	*Thymus serphyllum* L./RW755	Lamiaceae
Chenga	*Persicaria vivipara* (L.) Ronse Decr./RW756	Polygonaceae
Goom	*Triticum aestivum* L./RW757	Poaceae
Bushi punar	*Saussurea gossypiphora* D.Don/RW742	Compositae
Susar	*Rhododendron anthopogon* D. Don/RW758	Ericaceae
Patrees	*Aconitum heterophyllum* Wall. ex Royle/RW759	Ranunculaceae
Jomi	*Urtica dioica* L./RW760	Urticaceae
Koret	*Bergenia stracheyi* (Hook.f. & Thomson) Engl./RW761	Saxifragaceae
Simbul Char	*Adiantum raddianum C. Presl*/RW762	Pteridaceae
One	*Cucurbita maxima* Duchesne/RW763	Cucurbitaceae
Shatoo	*Ribes alpestre* Wall.ex Decne./RW764	Grossulariaceae
Margosh Chontal	*Rheum australe* D. Don/RW743	Polygonaceae
Peban Maroch	*Morus alba* L./RW765	Moraceae
Mulo	*Raphanus sativus* L./RW766	Brassicaceae
Gizari	*Daucus carota* L./RW767	Umbelliferae
Nooni Char	*Oxalis corniculata* L./RW768	Oxalidaceae
Chorko	*Berberis lycium* Royle/RW769	Berberidaceae
Konay	*Echinops echinatus* Roxb./RW770	Compositae
Churki	*Rumex hastatus* D. Don/RW771	Polygonaceae
Hailel	*Solanum nigrum* L./RW772	Solanaceae
Khakao	*Pistacia khinjuk* Stocks/RW773	Anacardiaceae
Khaneray Char	*Salvia* sp./RW737	Lamiaceae
Hamay	*Dysphania botrys* (L.) Mosyakin & Clemants/RW774	Amaranthaceae
Bhendi	*Abelmoschus esculentus* (L.) Moench/RW775	Malvaceae
Hayao	*Bunium persicum* (Boiss) B. Fedtsch./RW776	Apiaceae

Makai	*Zea mays* L./RW777	Poaceae
Tandur	*Datura stramonium* L./RW778	Solanaceae
Shangali	*Cuscuta reflexa* Roxb.	Convolvulaceae
Shaftal	*Trifolium repens* L./RW737	Leguminosae
Bangra	*Swertia petiolata* D. Don/RW738	Gentianaceae
Khapoy Patay	*Plantago himalaica* Pilg./RW779	Plantaginaceae
Chilli	*Juniperus excelsa* M.Bieb./RW780	Cupressaceae
Danoi	*Punica granatum L.*/RW781	Punicacea
Zooti Ponar	*Aster himalaicus* C. B. Clarke/RW782	Compositae
Kasheel/Zach	*Vitis vinifera* L./RW783	Vitaceae
Gulab	*Rosa indica* L./RW784	Rosaceae
Kuna	*Chenopodium album* L./RW785	Amaranthaceae
Aseel Khukunay	*Cicer microphyllum* Benth./RW786	Leguminosae
Loi Margan	*Capparis spinosa* L./RW787	Capparaceae
Kino Maroch	*Morus nigra* L./RW788	Moraceae
Hazar Daru	*Limonium cabulicum* (Boiss.) Kuntze/RW789	Plumbaginaceae
Dadi Pushi Char	*Xanthium strumarium* L./RW790	Compositae
Pharphara	*Verbascum thapsus* L./RW791	Scrophulariaceae
Ishpit	*Medicago sativa* L./RW792	Leguminosae
Khakos	*Artemisia annua* L./RW793	Compositae

Uses	Classification	Used form	Occurrence	Availability
Intestinal worms (65),	Angiosperm	Fresh	Foothills,	Frequent
diarrhea (89), intestina	Angiosperm	Fresh & dried	Mountains, plains	Frequent
diarrhea (89), vomiting	Angiosperm	Fresh	Foothills, mountains, p	Occasional
Diarrhea (79), indigest	Angiosperm	Fresh & dried	Mountains	Frequent
Diarrhea (51), vomitin	Angiosperm	Fresh	Foothills/mountains/pl	Frequent
Stomachic (42), Intest	Angiosperm	Dried	Plains	Rare
Constipation (132)	Angiosperm	Dried	Mountains	Rare
Stomachic (31), consti	Angiosperm	Dried	Mountains	Occasional
diarrhea (67) & digest	Angiosperm	Fresh	Mountains	Frequent
diarrhea (23), constipa	Angiosperm	Fresh	Mountains	Occasional
Stomach disorders (57	Angiosperm	Dried	Mountains	Occasional
Ulcer (22), stomachic	Angiosperm	Fresh	Mountains	Frequent
Intestinal worms (21),	Angiosperm	Fresh	Plains	Frequent
Constipation (24) & st	Angiosperm	Fresh	Foothills/plains	Rare
Diarrhea (32) & consti	Angiosperm	Fresh & dried	Plains	Frequent
Digestive (29) & purg	Angiosperm	Fresh	Foothills/plains	Occasional
Stomachic (75)	Angiosperm	Fresh & dried	Foothills/plains	Frequent
diarrhea (3) & constip	Angiosperm	Fresh	Mountains	Occasional
Piles (56)	Angiosperm	Dried	Mountains	Frequent
constipation (54)	Angiosperm	Dried	Foothills/plains	Rare
Stomachic (74)	Angiosperm	Fresh & dried	Mountains	Occasional
Stomachic (27) & inte	Angiosperm	Dried	Mountains	Rare
Vomiting (31)	Angiosperm	Dried	Mountains	Occasional
Stomachic (47) & diar	Angiosperm	Dried	foothills/mountain	Frequent
diarrhea (41)	Pteridophytes	Dried	Mountains	Occasional
Stomachic (27) & con	Angiosperm	Fresh	Plains	Occasional
Ulcer (22)	Angiosperm	Fresh	Foothills/plains	Rare
Intestinal worms (64)	Angiosperm	Dried	Mountains	Rare
Stomachic (29) & con	Angiosperm	Fresh & dried	Mountains	Frequent
Stomachic (33)	Angiosperm	Fresh	Foothills/plains	Occasional
Digestive (43)	Angiosperm	Fresh	Foothills/plains	Occasional
Constipation (23) & ul	Angiosperm	Fresh	Foothills/plains	Occasional
Stomach problem (41)	Angiosperm	Dried	Plains	Rare
Abdominal pain (22)	Angiosperm	Fresh	foothills/mountain	Occasional
Stomachic (29) & flatu	Angiosperm	Fresh	Foothills	Frequent
Dysentery (9), stomac	Angiosperm	Fresh	Foothills/plains	Occasional
indigestion (41)	Angiosperm	Fresh & dried	Foothills/plains	Occasional
Stomachic (71)	Angiosperm	Dried	foothills/mountain	Rare
Abdominal pain (6) &	Angiosperm	Fresh	Mountains	Occasional
Digestive (27)	Angiosperm	Fresh	Foothills/plains	Frequent
stomachic (41)	Angiosperm	Fresh & dried	Foothills/plains	Rare

Stomachic (32) & gast	Angiosperm	Dried	Mountains	Frequent
Stomachic (32)	Angiosperm	Dried	Foothills/plains	Rare
Stomach disorders (23	Angiosperm	Fresh	Plains	Occasional
Stomachic (22)	Angiosperm	Fresh	Plains	Occasional
Stomachic (41)	Angiosperm	Dried	Plains	Frequent
Stomachic (19) & diar	Angiosperm	Fresh	Mountains	Frequent
diarrhea (43)	Gymnosperms	Fresh & dried	Plains	Frequent
Intestinal worms (32)	Angiosperm	Fresh & dried	foothills/mountain	Frequent
Stomachic (18)	Angiosperm	Fresh & dried	foothills/plains	Occasional
Constipation (37) & in	Angiosperm	Fresh & dried	Mountains	Frequent
Stomachic (19) & cons	Angiosperm	Fresh	Foothills/plains	Occasional
Constipation (12) & ab	Angiosperm	Fresh	Foothills/plains	Occasional
Indigestion (5) & vom	Angiosperm	Fresh	Foothills/plains	Occasional
Piles (7) & digestive (	Angiosperm	Dried	Mountains	Frequent
Stomachic (21)	Angiosperm	Fresh & dried	Plains	Frequent
Stomachic (21)	Angiosperm	Dried	Foothills/plains	Rare
Ulcer (11)	Angiosperm	Fresh	Mountains	Rare
diarrhea (11)	Angiosperm	Fresh	Plains	Rare
Stomachic (12)	Angiosperm	Fresh	Mountains	Occasional
diarrhea (3) & vomitin	Angiosperm	Fresh	Foothills/plains	Occasional
			foothills	

Life form	Part(s) used	dministration rou	Mode of utilization
Herb	Leaves	Oral	Juice, paste
Herb	Leaves	Oral	Powder, juice, paste
Herb	Leaves	Oral	Juice
Herb	Leaves	Oral	Powder, juice, paste
Herb	Bulb, leaves	Oral	Juice, paste
Herb	Root	Oral	Powder
Herb	Whole	Oral	Powder
Herb	Root	Oral	powder
Herb	Leaves	Oral	Direct
Herb	Leaves, fruit	Oral	Direct
Herb	Root	Oral	Powder
Herb	Aerial parts	Oral	Juice, paste
Tree	Leaves, fruit	Oral	Direct, juice
Tree	Fruit	Oral	Direct
Tree	Fruit	Oral	Direct
Herb	Fruit	Oral	Direct
Herb	Leaves	Oral	Decoction
Herb	Fruit	oral	Direct
Herb	Fruit	Oral	Powder, roast
Herb	Flower	Oral	Decoction
Shrub	Leaves, flower	Oral	Decoction
Herb	Root	Oral	Powder
Herb	Leaves, root	Oral	Powder
Herb	Root	Oral	Powder
Herb	Leaves	Oral	Powder
Herb	Fruit	Oral	Roast
Shrub	Fruit	Oral	Direct
Herb	Root	Oral	Powder
Tree	Fruit	Oral	Direct, powder
Herb	Root	Oral	Direct
Herb	Root	Oral	Direct
Herb	Leaves	Oral	Direct
Herb	Bark	Oral	Infusion, Powder
Herb	Leaves	Oral	Infusion
Herb	Aerial parts	Oral	Juice
Herb	Fruit	Oral	Direct
Tree	Fruit, gum	Oral	Powder, direct
Herb	Root	Oral	Powder
Herb	Leaves	Oral	Direct
Herb	Fruit	Oral	Roast
Herb	Seed	Oral	Decoction

Herb	Fruit	Oral	Powder, roast
Herb	Seed	Oral	Powder
Herb	Whole	Oral	Roast
Herb	Leaves	Oral	Roast
Herb	Aerial parts	Oral	Powder, infusion
Herb	Leaves	Oral	Juice, paste
Tree	Leaves, fruit	Oral	Powder, smoke
Tree	Fruit, exocarp of fruit	Oral	Powder
Herb	Flower	Oral	Powder
	Leaves, fruit	Oral	Direct
Shrub	Flower	Oral	Powder
Herb	Leaves	Oral	Direct
Herb	Seed	Oral	Direct
Herb	Seed	Oral	Powder
Tree	Fruit	Oral	Direct, powder
Herb	Whole	Oral	Powder
Herb	Leaves	Oral	Paste
Herb	Leaves	Oral	Juice
Herb	Leaves	Oral	Roast
Herb	Leaves	Oral	Paste

Method of preparation	UR	Use citations	FC	RFC	UV	N
Fresh leaves mixed with mint, grinded ir	4	200	112	0.67	1.79	166
Fresh and dried leaves grinded into paste	5	207	140	0.84	1.48	
Fresh leaves grinded with mint leaves ar	3	189	132	0.80	1.43	
Fresh and dried leaves grinded into paste	3	109	99	0.60	1.10	
Fresh leaves and bulb is grinded with lea	2	85	78	0.47	1.09	
One teaspoon of dried root powder is mi	2	80	86	0.52	0.93	
Half a teaspoon of dried powder or 5-10	1	132	143	0.86	0.92	
one to two teaspoons of dried root powd	3	70	76	0.46	0.92	
Fresh leaves are taken directly	2	103	112	0.67	0.92	
Fresh leaves and fruit taken orally	3	57	62	0.37	0.92	
Dried root powder of the plant is mixed	1	57	62	0.37	0.92	
fresh aerial parts of the plant are grinded	2	65	79	0.48	0.82	
Fresh fruits are taken directly while 300-	3	56	69	0.42	0.81	
Fresh and dried fruit taken orally	2	55	68	0.41	0.81	
Fresh and dried fruit of the plant taken o	2	53	67	0.40	0.79	
Fresh fruit is taken orally	2	59	77	0.46	0.77	
Decoction of fresh and dried leaves is us	1	75	98	0.59	0.77	
Fresh and dried fruit is used orally	2	46	61	0.37	0.75	
Dried fruit is grinded, mixed with water	1	56	76	0.46	0.74	
Decoction of the dried flower is taken da	1	54	75	0.45	0.72	
Decoction of fresh and dried flower and	1	74	104	0.63	0.71	
Half teaspoon of the root powder is mixe	2	43	61	0.37	0.70	
Dried leaf and root powder is mixed wit	1	31	44	0.27	0.70	
Two to three teaspoon of dried root pow	2	71	102	0.61	0.70	
Dried leaf powder is taken orally	1	41	59	0.36	0.69	
Fresh fruit is cooked and taken orally	2	43	62	0.37	0.69	
fresh fruit of the plant used directly	1	22	32	0.19	0.69	
One teaspoon of dried root powder mixe	2	96	142	0.86	0.68	
Fresh and dried fruit is used orally	2	50	74	0.45	0.68	
Fresh root of the plant is used directly	1	33	50	0.30	0.66	
Fresh root of the plant is used directly	1	43	67	0.40	0.64	
Fresh leaves taken orally	2	41	65	0.39	0.63	
Bark of the plant is shade dried and grin	1	41	65	0.39	0.63	
Infusion of fresh leaves is used orally	1	22	35	0.21	0.63	
Fresh aerial parts are grinded with water	2	51	82	0.49	0.62	
Fresh fruit is taken orally	3	40	65	0.39	0.62	
Powder of the fruit is taken with water w	1	41	67	0.40	0.61	
Dried root powder of the plant is mixed	1	71	118	0.71	0.60	
Fresh leaves are taken directly	2	23	41	0.25	0.56	
Roasted fruit is used in the form of vege	1	27	49	0.30	0.55	
Decoction of the dried seeds is used at b	1	41	76	0.46	0.54	

Dried fruit is grinded, mixed with water	2	59	111	0.67	0.53
one to two teaspoons of dried seed powd	1	32	61	0.37	0.52
Fresh plant is collected and used along w	1	23	44	0.27	0.52
Fresh leaves of the plant used as a veget	1	22	43	0.26	0.51
Dried aerial parts of the are used in the f	1	41	81	0.49	0.51
Juice and paste of fresh leaves is taken o	2	28	59	0.36	0.47
Smoke of the leaves of the plant is inhal	1	43	92	0.55	0.47
Fresh and stored fruit is taken orally whi	1	32	72	0.43	0.44
Fresh flowers are collected, dried, grind	1	18	44	0.27	0.41
Fresh leaves and fruit are used orally	2	40	98	0.59	0.41
Dried flower powder is mixed with wate	2	31	78	0.47	0.40
Fresh leaves are taken directly	2	17	44	0.27	0.39
Fresh seed are taken orally	2	14	39	0.23	0.36
one teaspoon of shade dried seed powde	2	12	35	0.21	0.34
Fresh and dried fruit are used in the forn	1	21	63	0.38	0.33
Dried powder of the plant is mixed with	1	21	75	0.45	0.28
Paste of the fresh leaves is taken orally	1	11	43	0.26	0.26
Freshly collected leaves of the plant is g	1	11	54	0.33	0.20
Fresh and young leaves of the plant are r	1	12	65	0.39	0.18
Paste of the fresh leaves is taken orally	2	7	58	0.35	0.12

Male	Femal
115	51
