# Supplementary material for: Ethnomedicinal appraisal of plants used for the treatment of gastrointestinal complaints by tribal communities living in Diamir district, Western Himalayas, Pakistan
Source: PLoS One. 2022 Jun 8;17(6):e0269445. doi: 10.1371/journal.pone.0269445 (PMC9176800; doi:10.1371/journal.pone.0269445)
Supplement: S2 Table — (PDF) [file pone.0269445.s002.pdf]

Disease
Stomachic
Diarrhea

Constipation

Vomiting

Intestinal worms

Gastritis

Indigestion

Digestive

Piles

Ulcer

Flatulence

Purgative

Abdominal pain

Dysentary

Species

Artemisia maritima L.

Artemisia maritima L.

Mentha longifolia (L.) L.

Mentha piperita L.

Allium cepa L.

Artemisia annua L.

Hylotelephium telephioides (Ledeb.) H. Ohba

Artemisia maritima L.

Mentha longifolia (L.) L.

Allium cepa L.

Tanacetum faconeri Hook.f.

Artemisia annua L.

Mentha longifolia (L.) L.

Thymus serphyllum L.

Vitis vinifera L.

Mentha longifolia (L.) L.

Berberis lycium Royle

Bergenia stracheyi (Hook.f. & Thomson) Engl.

Rhododendron anthopogon D. Don

Swertia petiolata D. Don

Tanacetum faconeri Hook.f.

Aconitum heterophyllum Wall. ex Royle

Cichorium intybus L.

Artemisia maritima L.

Cucumis sativus L.

Tanacetum faconeri Hook.f.

Bunium persicum (Boiss) B. Fedtsch.

Pimpinella diversifolia DC.

Abelmoschus esculentus (L.) Moench

Cichorium intybus L.
Prunus armeniaca L.
Zea mays L.
Pimpinella diversifolia DC.
Ficus carica L.
Mentha piperita L.
Plantago himalaica Pilg.
Rumex hastatus D. Don
Datura stramonium L.
Mentha longifolia (L.) L.
Salvia sp.
Bergenia stracheyi (Hook.f. & Thomson) Engl.
Juniperus excelsa M.Bieb.
Morus alba L.
Rheum webbianum Wall.
Daucus carota L.
Triticum aestivum L.
Ficus carica L.
Rheum australe D. Don
Prunus persica L.
Prunus armeniaca L.
Persicaria amplexicaulis (D.Don) Ronse Decr.
Rheum webbianum Wall.
Capparis spinosa L.
Mentha piperita L.
Limonium cabulicum (Boiss.) Kuntze
Pistacia khinjuk Stocks
Zea mays L.
Viola serpens WalL.
Oxyria digyna (L.)Hill

Cicer microphyllum Benth.
Rheum australe D. Don
Rumex hastatus D. Don
Rosa indica L.
Prunus persica L.
Cucumis sativus L.
Aster himalaicus C. B. Clarke
Cucurbita maxima Duchesne
Trifolium repens L.
Solanum nigrum L.
Urtica dioica L.
Oxyria digyna (L.)Hill
Rheum webbianum Wall.
Morus nigra L.
Cuscuta reflexa Roxb.
Cucurbita maxima Duchesne
Viola serpens WalL.
Solanum nigrum L.
Verbascum thapsus L.
Cicer microphyllum Benth.
Echinops echinatus Roxb.
Adiantum raddianum C. Presl
Aconitum heterophyllum Wall. ex Royle
Prunus persica L.
Dysphania botrys (L.) Mosyakin & Clemants
Morus alba L.
Plantago himalaica Pilg.
Raphanus sativus L.
Oxalis corniculata L.
Ribes alpestre Wall.ex Decne.

Solanum nigrum L.
Medicago sativa L.
Rosa indica L.
Capparis spinosa L.
Persicaria vivipara (L.) Ronse Decr.
Dysphania botrys (L.) Mosyakin & Clemants
Vitis vinifera L.
Oxalis corniculata L.
Punica granatum L.
Persicaria vivipara (L.) Ronse Decr.
Xanthium strumarium L.
Chenopodium album L.
Saussurea gossypiphora D.Don
Chenopodium album L.

**Ip**	**Iu**	**FL(%)**
50	50	100.0
57	57	100.0
66	66	100.0
54	54	100.0
57	57	100.0
43	43	100.0
56	56	100.0
56	56	100.0
51	51	100.0
47	47	100.0
45	47	95.7
42	45	93.3
49	53	92.5
45	51	88.2
45	53	84.9
48	57	84.2
34	41	82.9
41	50	82.0
43	53	81.1
38	48	79.2
33	42	78.6
33	42	78.6
33	43	76.7
41	54	75.9
34	45	75.6
34	46	73.9
31	42	73.8
29	41	70.7
34	52	65.4

28	43	65.1
33	53	62.3
23	42	54.8
31	57	54.4
23	44	52.3
24	46	52.2
21	41	51.2
22	43	51.2
22	43	51.2
23	45	51.1
23	47	48.9
23	47	48.9
23	47	48.9
23	47	48.9
21	43	48.8
21	44	47.7
21	44	47.7
21	45	46.7
21	46	45.7
18	41	43.9
23	54	42.6
19	45	42.2
21	54	38.9
20	52	38.5
21	55	38.2
17	45	37.8
17	46	37.0
21	57	36.8
15	43	34.9
15	43	34.9

15	45	33.3
17	52	32.7
15	49	30.6
14	46	30.4
17	56	30.4
17	56	30.4
13	43	30.2
13	43	30.2
8	27	29.6
13	44	29.5
13	44	29.5
13	45	28.9
13	45	28.9
15	52	28.8
11	39	28.2
11	41	26.8
11	41	26.8
11	41	26.8
11	42	26.2
11	43	25.6
11	43	25.6
11	44	25.0
11	45	24.4
11	45	24.4
8	34	23.5
13	56	23.2
7	31	22.6
9	41	22.0
9	41	22.0
9	43	20.9

11	53	20.8
9	46	19.6
8	45	17.8
9	51	17.6
7	41	17.1
8	47	17.0
9	55	16.4
7	43	16.3
9	56	16.1
6	39	15.4
7	46	15.2
6	41	14.6
7	54	13.0
5	51	9.8
