# Supplementary material for: Ethnomedicinal appraisal of plants used for the treatment of gastrointestinal complaints by tribal communities living in Diamir district, Western Himalayas, Pakistan
Source: PLoS One. 2022 Jun 8;17(6):e0269445. doi: 10.1371/journal.pone.0269445 (PMC9176800; doi:10.1371/journal.pone.0269445)
Supplement: Attachment — Submitted filename: Response to Reviewer.docx [file pone.0269445.s003.docx]

**Response to the reviewer’s comments**

|  |  | Response to reviewer 1 |  |
| --- | --- | --- | --- |
| S. No | Reviewer | Comment | Response |
| 1 | R1 | Toxicity or other adverse effects of the plant would have been noted  Exact dose of the *Datura stramonium* needs to be determined. | Our survey was somehow targeted to gather plant use information specially for the treatment of GIDs that’s why information on toxicity or adverse effects was not recorded however most of the non-practicing informants and all of the herbal practitioners stressed on use of botanicals in very quantities and this information is well recorded in method of preparation section for example the dose of *Datura stramonium “*one to two teaspoons of dried seed powder are mixed with 500 ml of water and taken once a week*”* (Supplementary file 1; row 44, Column M) |
| 2 | R1 | Need information regarding the use of plant part(s) in the survey area versus other reported areas. | We have mentioned the novel use report(s) from the survey area and compared it with already reported plant rather than comparing the plant part(s) used. The literature search works with plant name and hence it was considered less likely that a previous report is overlooked because of different part use information. However, we have carefully rechecked our novelty information and found that novelty of plant species was intact at least up to the part use reported here for a specific disorder |
| 3 | R1 | No detailed information has been given on how the plants were collected; whether they were used in fresh or dried state; if dry then how long can the medicinal activities of a given plant be sustained. | The plants were collected in fresh form initially. Some of the plants were used in fresh form and some in dried form. Information incorporated in supplementary file 1. Mostly locals use the dried plants within 1-2 years of duration. |
| 4 | R1 | Preparation of medicine like powder, decoction or other forms, dosage, part of plant used, use of monoherbal or polyherbal formulation. | Detailed Information regarding preparation method is given in Supplementary file 1 (Columns JKL& M) noteworthy information is also mentioned in figure 3(a). Regarding formulation like monoherbal and polyherbal, information mentioned in supplementary file 1. |
| 5 | R1 | If the same plant is used for more than one gastrointestinal disorder like say diarrhea and constipation did the mode of use differed and how? | Mode of use of same plant for more than one gastrointestinal disorder also mentioned in supplementary file 1. The mode of use is same for more than one disease treated as mentioned in supplementary file 1. |
| 6 | R1 | Endangerment of these medicinal plants and any efforts by the locals in their conservation. | As such locals do not practice any conservation efforts however local people understand that excessive use of plant part(s) can lead the plant for its endangerment, so the locals tend to reduce the overhunting of plant part(s). |
| 7 | R1 | The altitude range where the plants can be found. | The altitude range where the plants were collected is mentioned in Fig 1, which between 35°23'14.3"N latitude and 74°34'44.7"E longitude, at an elevation range between 934-7937 m above the sea level. |
| 8 | R1 | The authors note that elderly people possessed more knowledge of traditional plant usage. In that case, why were more elderly people not consulted to gather more information? That elderly people know more of their traditional customs is open knowledge; the question is how can the young generation be made more interested in the traditional customs? | The population in the study area is not so high and we know that number of elders in a population is always comparatively low. We tried to consult the elders of the survey area aged between 50-60 that comprised up of 27.1% and above 60 years 25.9%. Secondly, in the study area some of the elders don’t want to share their traditional knowledge as they think that their traditional knowledge may be stolen by others. Thirdly, there is health factor of the elders also involved, as the elders are weaker than the youngsters, so they don’t have much energy to discuss with the researcher for a longer discussion. |
|  |  | Response to reviewer 2 |  |
| 1 | R2 | ♣ The abstract is well-structured, well written and has excellently discussed and concluded the different aspects. However, it would be highly appreciated if authors incorporate sentence about the future perspectives of the research work conducted from present study. | Plant species with maximum medicinal values could be a potential source of novel drug leads to cure gastrointestinal disorders. |
| 2 | R2 | ♣ Add the word “The” before “resent study was conducted in fairy meadows” in the abstract. | Word “The” added as directed. |
| 3 | R2 | ♣ There is no future perspective statement in the abstract section. Provide a statement that what next can be done after these ethnobotanical surveys. | Two sentences added at the end of the abstract which are “This showed that occupants of the study area have sound information about ethno-pharmacological consumption of medicinal plants with some novel use reports which may provide the basic data for further pharmacological research. Plant species with maximum medicinal values could be a potential source of novel drug leads to cure gastrointestinal disorders” shows the future perspective statement in the abstract section. |
| 4 | R2 | ♣ The introduction section is quite informative and to the point. Authors have comprehensively discussed the notion behind present research work. Authors are advised once again to double check grammar, sentence structure etc, if there any deficiency, fix them accordingly. | All done. |
| 5 | R2 | ♣ Provide updated literature to the statement “According to world health organization (WHO) estimates…………. million deaths on 2012 alone. Update 2021-2022 statistics if available. | Updated as suggested. |
| 6 | R2 | ♣ There is no discussion on the overall medicinal plants flora of the said geographical area. Provide few words and discuss about the dominant medicinal plants. | Discussion added as suggested |
| 7 | R2 | ♣ What was ecological distribution and phytogeography, utilization status and threats to the surveyed medicinal plants? Do you have some recommendations regarding their conservation aspects? I think you must add 1-2 lines. | Added in results a separate subheading as “Phytogeographic distribution and utilization status of medicinal plants” recommendations regarding conservation of medicinal flora added in the conclusion |
| 8 | R2 | ♣ Results and discussion section is well written, all results have been rightly discussed with relevant data from literature. Add some new 2020-2021 data from literature if available. | Done as suggested |
| 9 | R2 | ♣ The conclusion section is comprehensive and well-articulated. Remove results and introductory statements from conclusion section after carefully reviewing manuscript. | Conclusion revised according to the suggestions |
| 10 | R2 | ♣ There is no uniformity in references. Cross check all the references and strictly follow PLOS ONE author guidelines. | All done. |
|  |  | Response to reviewer 3 |  |
| 1 | R3 | Authors must concentrate on the formatting, and use of symbols, etc., throughout the manuscript. | All done. |
| 2 | R3 | Continuous line numbers are needed for point out the syntax error, corrections in the manuscript. | Done |
| 3 | R3 | Keywords should be unbold. | Done. |
| 4 | R3 | In introduction, authors have cited the WHO report 2017 it should be referred and cite after 2019 WHO report is better. | Done as suggested |
| 5 | R3 | Authors should refer few more GIDs articles and include some lines in the introduction section. Because the GIDs information is looks shallow. Better to discuss deeply. | Updated as suggested |
| 6 | R3 | Institutional ethical committee number should be added in the statement of ethics section. | Ethical committee number added as UPR/HACE/01/13/21. Dated March 16, 2021. |
| 7 | R3 | Remove the dot in front of lines in Documentation of Ethnomedicinal Data section. | Done |
| 8 | R3 | In sub-title of Results and Discussion section should be “Results”. Because authors have written the separate discussion section. | Done |
| 9 | R3 | Framework figure is required. It will be useful to the readers for better understanding of the studied issue. | Framework figure added with the map of the study area (Fig.1). As there is a limit of 5 figures from PLOS at max. |
| 10 | R3 | In materials and method section: authors should explain why each item of methodology was done. | Explained |
| 11 | R3 | Proper formatting needed in the materials and methods, results, discussion, and conclusion section. | Done. |
| 12 | R3 | In conclusion section authors should provide some future prospectus related to present study. | Done as suggested |
| 13 | R3 | Reference section should be formatted according to the journal. | All done. |
| 14 | R3 | Authors should clearly state the figure caption as well as description for ease of understanding of the readers. | All done. |
